# Comprehensive analysis of lncRNA and mRNA based on expression microarray profiling reveals different characteristics of osteoarthritis between Tibetan and Han patients

**DOI:** 10.1186/s13018-021-02213-y

**Published:** 2021-02-12

**Authors:** Junming Luo, Xiaoqin Luo, Zhili Duan, Wenbin Bai, Xiaoming Che, Zhongshu Shan, Xiaona Li, Jinwu Peng

**Affiliations:** 1 Department of Pathology, Xiangya Changde Hospital, Changde, 415000 Hunan China; 2 Department of Pathology, Qinghai Provincial People’s Hospital, Xining, 810007 Qinghai China; 3 Department of Orthopedic Surgery, Qinghai Provincial People’s Hospital, Xining, 810007 Qinghai China; 4 Department of Pathology, Xiangya Basic Medical School, Central South University, Changsha, 410013 Hunan China; 5 Department of Pathology, Xiangya Hospital, Central South University, Changsha, 410008 Hunan China

**Keywords:** Osteoarthritis, Tibetan, lncRNA-mRNA, Microarray profiling, OTOA

## Abstract

**Background:**

Osteoarthritis (OA) is thought to be the most prevalent chronic joint disease, especially in Tibet of China. Here, we aimed to explore the integrative lncRNA and mRNA landscape between the OA patients of Tibet and Han.

**Methods:**

The lncRNA and mRNA expression microarray profiling was performed by SurePrint G3 Human Gene Expression 8x60K v2 Microarray in articular cartilage samples from OA patients of Han nationality and Tibetans, followed by GO, KEGG, and trans-regulation and cis-regulation analysis of lncRNA and mRNA.

**Results:**

We found a total of 117 lncRNAs and 297 mRNAs differently expressed in the cartilage tissues of Tibetans (*n* = 5) comparing with those of Chinese Han (*n* = 3), in which 49 lncRNAs and 158 mRNAs were upregulated, and 68 lncRNAs and 139 mRNAs were downregulated. GO and KEGG analysis showed that several unreported biological processes and signaling pathways were particularly identified. LncRNA-mRNA co-expression analysis revealed a remarkable lncRNA-mRNA relationship, in which OTOA may play a critical role in the different mechanisms of the OA progression between Tibetans and Chinese Han.

**Conclusion:**

This study identified different lncRNA/mRNA expression profiling between OA patients of Tibetans and Han, which were involved in many characteristic biological processes and signaling pathways.

**Supplementary Information:**

The online version contains supplementary material available at 10.1186/s13018-021-02213-y.

## Introduction

Osteoarthritis (OA) is the most common skeletal disease worldwide, causing pain, dyskinesia for patients, and a severe burden to society [1]. The incidence of osteoarthritis is rising because of the aging population and the epidemic of obesity [2]. Pain and loss of function are the main clinical characteristics that lead to treatment containing non-pharmacological, pharmacological, and surgical approaches [3]. Clinicians recognize that the diagnosis of osteoarthritis may be too late to expect much help from disease-modifying drugs [4]. Despite efforts over the past decades to excavate markers of disease, still-imaging procedures and biochemical marker analyses need to be improved and possibly extended with more specific and sensitive methods to reliably describe disease processes, to diagnose the disease earlier, and to follow the course of disease and treatment effectiveness [5, 6]. Hence, the investigation and exploration of the biological mechanism and biomarkers of OA are critical to understanding the OA pathophysiology. China is the country with the largest incidence of OA, such as Kashin Beck Disease (KBD) in the world, and the Tibetans are the people most affected by OA in China [7]. In particular, in the Qing-Tibet Plateau, a selenium-deficient region, the prevalence of OA is serious and still increasing and continues to damage public health for Tibetans [8]. More importantly, due to the specific climate and living environment and life habits in Tibet, it exhibits particular characteristics of OA between Tibetans and other populations, including the Han nationality [9]. In Tibet, where severe selenium deficiency is endemic, iodine deficiency is a risk factor for OA [10]. Besides, the long-term or severe cold stimulation and the mutual labor weaken the metabolism and immune defense of articular cartilage, leading to cartilage surface ulceration and damage inflammation of OA patients in Tibet [11, 12]. Thus, the investigation of the genetic and biological difference in OA progression between Tibetans and the Han nationality will benefit in understanding the different mechanisms of the development of OA between Tibetans and Chinese Han. However, the exploration in this field is still extremely limited.

Over the last decade, the development of high-throughput technologies has allowed an in-depth examination of the non-coding genome with unprecedented resolution and scale, in which long non-coding RNAs (lncRNAs) play crucial roles in many physiological and pathological processes [13]. But their role in biological function and the signaling pathway involved in OA remain unknown. It has been reported that lncRNAs are expressed aberrantly in OA [14]. Current evidence indicates that lncRNAs not only serve as positive or negative regulators of OA but also crosstalk with multiple potential targets to impact the critical events in the OA process [15, 16]. The identification of lncRNAs in OA patients reveals its diagnostic and therapeutic value [17–19]. However, the comprehensive landscape of lncRNA in OA development remains unclear. Besides, aberrant mRNA expression provides us with an opportunity to better diagnose and stratify the OA via established panels or discover new approaches [20]. Analysis of mRNA expression identifies multiple markers in OA patients and may be the key contributing factor to OA [21]. Gene expression microarray has been the primary biomarker platform ubiquitously applied in biomedical research, leading to enormous data, predictive models, and biomarkers accrued [22, 23]. Genetic profiling of the mRNA and lncRNA expression can be used for staging OA at the molecular level [24]. However, the comprehensively integrated analysis of lncRNA and mRNA in OA of Tibetans and Chinese Han based on gene expression profiling remains unreported.

In the present study, we performed genome-wide screening for mRNA and lncRNA profiles in OA patients of Tibetans and Han nationality. Our study aimed to characterize the different features of lncRNA and mRNA expression between Tibetans and Han nationality. We also focused on determining the biological processes and pathways that exhibited significant changes in this event. Strikingly, we identified OTOA as a critical factor among the interaction of lncRNAs and mRNAs in the different lncRNA-mRNA networks of OA development between Tibetans and Chinese Han. Our comprehensive analysis will provide valuable information for understanding the variant molecular mechanisms in the OA progression of Tibetans and Han nationality.

## Materials and methods

### Clinical cartilage sample collection

A total of 8 human articular cartilage samples were obtained from consented patients undergoing joint replacement surgery due to OA, including 3 Han nationality patients and 5 Tibetan patients. All the samples were snapped frozen in liquid nitrogen within 2 h of surgery and stored at 80 °C before RNA extraction. All cartilage was washed extensively in phosphate-buffered saline (PBS) to avoid contamination. All study subjects provided informed consent before the inclusion of people in the study. The study protocol was approved by the Qinghai Provincial People’s Hospital, China.

### RNA extraction

The processes of cartilage harvest, sectioning, grinding, and RNA extraction were performed as previously described [25, 26]. Briefly, articular cartilage samples were sectioned and powdered under liquid nitrogen; 100 mg of articular cartilage powder was used for RNA isolation with 5 ml of Trizol (Invitrogen, CA). The RNA concentration and quality (RNA integrity number (RIN) and 28S/18S ratio) were determined by a NanoDrop (NanoDrop Technologies, DE) and the RNA 6000 Nano Assay on an Agilent 2100 Bioanalyzer (Agilent Technologies, CA), respectively.

### LncRNA and mRNA microarray expression profiling

The lncRNA and mRNA microarray expression profiling was performed in articular cartilage samples from OA patients of Han nationality and Tibetans. Briefly, total RNA (100 ng) was labeled with the mRNA Complete Labeling and Hyb Kit (Agilent Technologies) and hybridized on the SurePrint G3 Human Gene Expression 8x60K v2 Microarray (Agilent Technologies). The microarray contains 50,599 probes for 32,776 human mRNA and 17,438 human lncRNAs, which are derived from authoritative databases, including RefSeq, Ensemble, GenBank, and the Broad Institute. After hybridization and washing, processed slides were scanned with the Agilent G2505C microarray scanner (Agilent Technologies). Raw data were extracted using Feature Extraction (version10.7.1.1; Agilent Technologies). Next, quantile normalization and subsequent data processing were done through the Genespring software (version 12.0; Agilent Technologies). The microarray profiling was conducted in Beijing Capitalbio Technology company.

### The analysis of differential expression level of mRNA and lncRNA from microarray

The differential expression level of mRNA and lncRNA from microarray was analyzed by using a limma R package. After quantile normalization, raw signals from microarray were log2 transformed. Differential expression of an mRNA or lncRNA was defined by absolute value of fold change (FC) > 2 (|log2FC| > 1) and *P* value < 0.05 (Student’s *t* test).

The differentially expressed mRNAs were submitted to the DAVID database (https://david.ncifcrf.gov) to be classified into different Gene Ontology [22] including molecular function (MF), biological process (BP) and cellular component (CC), and Kyoto Encyclopedia of Genes and Genomes (KEGG) annotation groups.

### LncRNA-mRNA co-expression analysis

The differential expression level of mRNA and lncRNA from microarray was analyzed by using a limma R package. After quantile normalization, raw signals from microarray were log2 transformed. Differential expression of an mRNA or lncRNA was defined by absolute value of fold change (FC) > 2 (|log2FC| > 1) and *P* value < 0.05 (Student’s *t* test).

The relationship between the differently expressed lncRNAs and mRNAs in Tibetan OA samples was assessed by Pearson correlation analysis. We calculated the Pearson correlation coefficient (PCC) of differentially expressed lncRNAs and mRNAs to unearth the co-expressed lncRNA and mRNAs. The absolute value of PCC > 0.7, as well as *P* < 0.05, was considered statistically significant.

Based on the co-expression, we further searched for the mechanism of how these aberrant lncRNAs realize functions through cis- or trans-regulating mRNAs. When the correlation of lncRNAs and mRNAs was significant (PCC > 0.7) and the mRNA loci were within 300 k windows up- and downstream of the given lncRNAs, we identified them as cis-regulated mRNAs of the corresponding lncRNAs. LncRNAs involved in certain biological pathways may interact with transcription factors (TFs) [27]. Thus, we performed the trans-regulation analysis to identify the co-expressed mRNAs significantly related to the dysregulated lncRNAs by using a threshold (PCC > 0.7, distance larger than 300 k) based on the Cytoscape software.

## Results

### OA clinical samples of Tibetans demonstrate significantly altered lncRNA and mRNA expression patterns compared with that of Han nationality

**Fig. 1:**
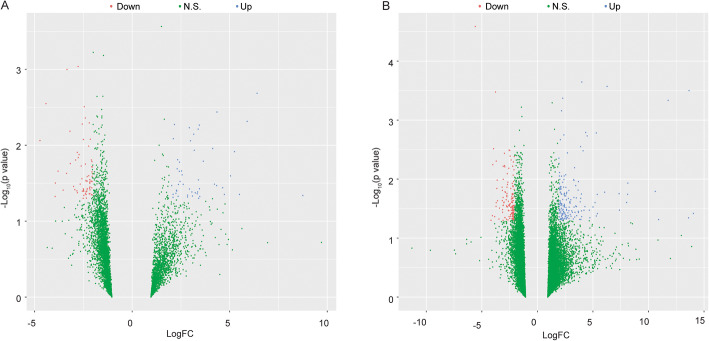
OA clinical samples of Tibetans demonstrate significantly altered lncRNA and mRNA expression patterns compared with that of Han nationality. **a**, **b** Volcano plot filtering map showed different expressions of lncRNA **a** and mRNA **b** in the cartilage tissues of Tibetans (*n* = 5) comparing with those of Chinese Han (*n* = 3). Blue and red represented upregulated or downregulated lncRNAs and mRNAs respectively, and green represented no significance. The x-axis was the Log2 fold change (FC) and the y-axis was − log10 (*p* value)

It has been reported that the development of OA in Tibet is prevalent, and many risk factors are involved in the OA progression of Tibetans [28]. Several lncRNAs have now been identified as being either differentially expressed in OA tissue or as candidate central regulators for OA progression [29]. Previous studies highlighted the importance of aberrant expression of lncRNA and mRNA in both the pathogenesis and potential treatment of OA [30]. Besides, the microarray analysis is an effective tool for the investigation of OA [31, 32]. Hence, we performed gene expression profiling based on the microarray analysis in the articular cartilage tissues of Tibetans and the Chinese Han. Interestingly, the volcano plot filtering has found total 117 lncRNAs and 297 mRNAs differently expressed in the cartilage tissues of Tibetans (*n* = 5) comparing with those of Chinese Han (*n* = 3), in which 49 lncRNAs and 158 mRNAs were upregulated in Tibetan samples (|log2 fold change | > 1, and *p* < 0.05) (Fig. 1 a and b). On the other hand, 68 lncRNAs and 139 mRNAs were found to be downregulated in the cartilage tissues of Tibetans (*n* = 5) comparing with those of Chinese Han (*n* = 3) (fold change > 2, and *P* < 0.05) (Fig. 1 a and b), suggesting that the different contributed factors are involved in the development of OA between Tibetans and the Han nationality.

### Many key genes and pathways are involved in the variant pathogenesis of OA between Tibetans and Chinese Han

**Fig. 2:**
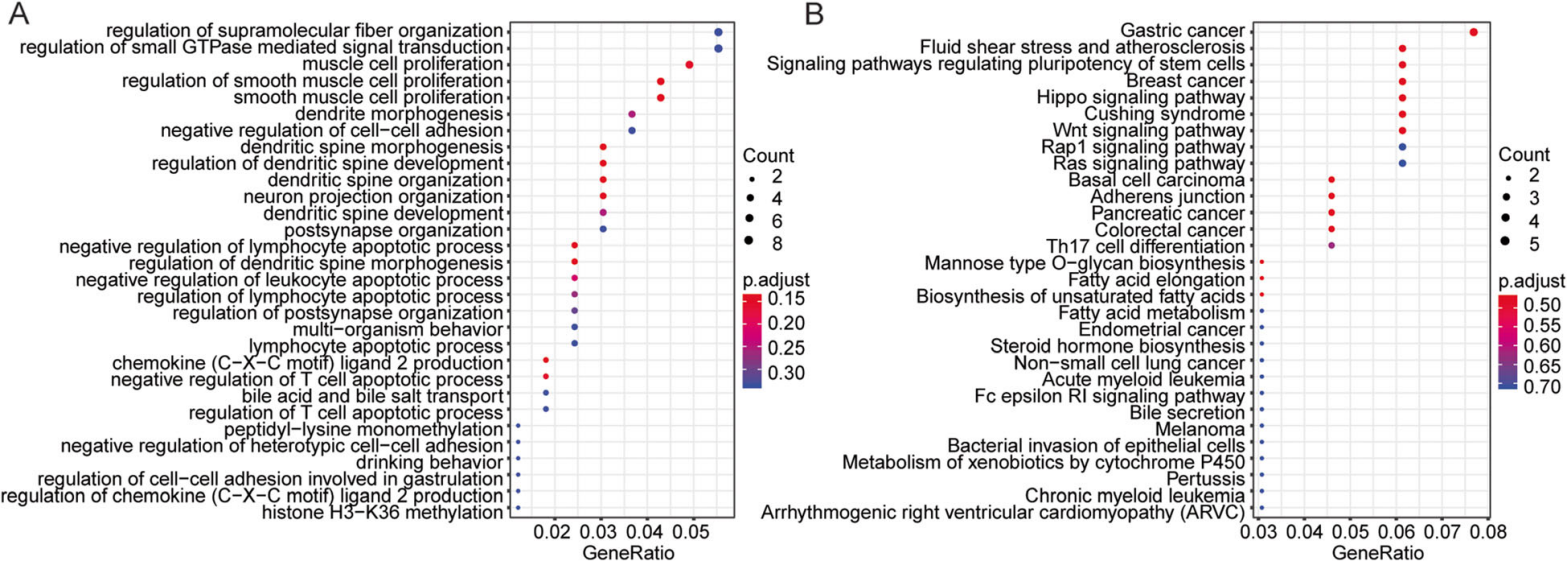
Many key genes and pathways are involved in the variant pathogenesis of OA between Tibetans and Chinese Han. **a**, **b** The Gene Ontology (GO) and Kyoto Encyclopedia of Genes and Genomes (KEGG) analysis were performed by using the data of differently expressed mRNA in the cartilage tissues of Tibetans (*n* = 5) comparing with those of Chinese Han (*n* = 3) based on the DAVID database (https://david.ncifcrf.gov). The top 30 significant cellular processes and signaling pathways were demonstrated by GO **a** and KEGG **b** enrichment scatter plot. The y-axis was the name of cellular processes or signaling pathways, and the x-axis was the gene ratio. The size of the dot revealed the number mRNAs

To further explore the potential function of the differently expressed genes, we performed Gene Ontology [22] and Kyoto Encyclopedia of Genes and Genomes (KEGG) analysis based on the above 297 identified mRNAs. Surprisingly, GO analysis revealed that the functions of these mRNAs were related to many important processes such as muscle cell proliferation, cell-cell adhesion, and chemokine ligand 2 production (Fig. 2 a and Table S1), implying that the different cellular processes may play specific roles in OA progression of Tibetans and Chinese Han. Significantly, KEGG analysis demonstrated that these mRNAs participated in several key signaling pathways, including the Hippo signaling pathway, Wnt signaling pathway, and Ras signaling pathway (Fig. 2b and Table S2), indicating the particular landscape of signaling pathway of OA in Tibetan and Han nationality.

### OTOA plays a crucial role in the different lncRNA-mRNA networks of OA progression between Tibetan and Chinese Han

**Fig. 3:**
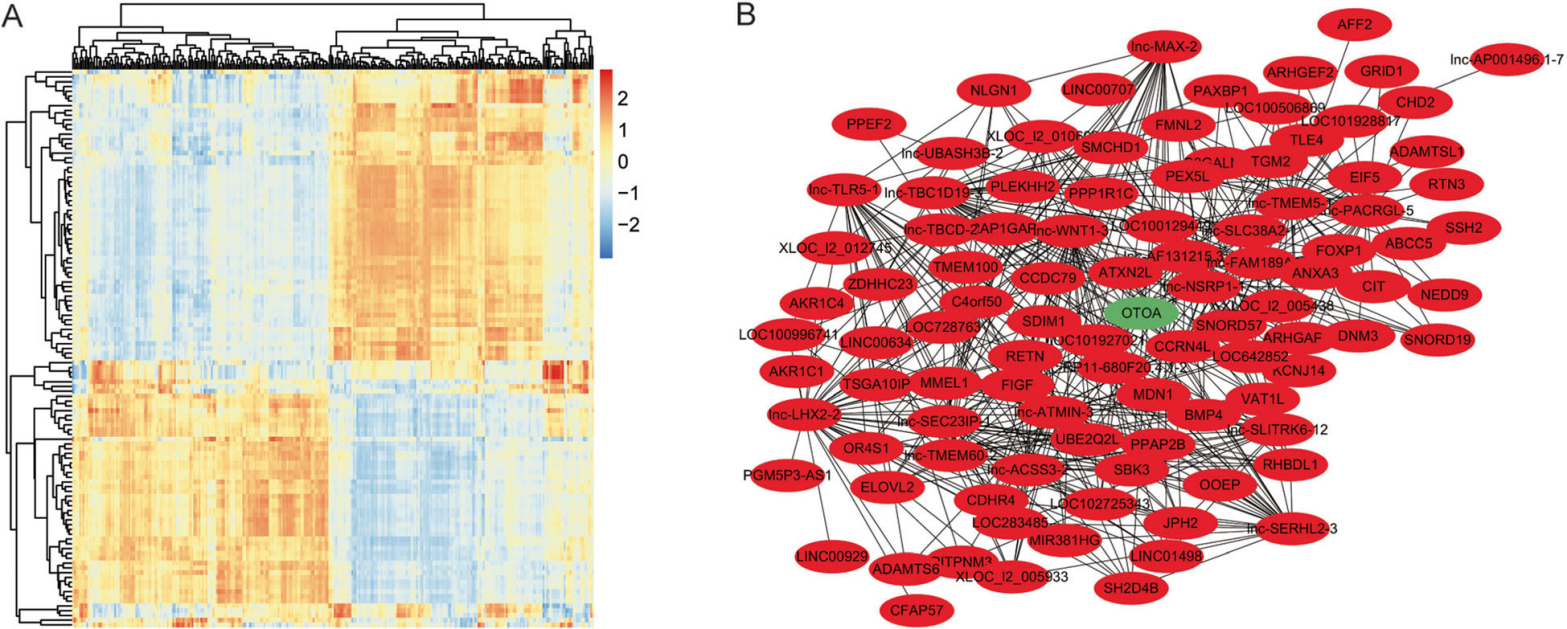
LncRNA-mRNA co-expression and trans-regulation analysis identify that OTOA plays a crucial role in the variant lncRNA-mRNA networks of OA progression in Tibetan and Chinese Han. **a** The heat map showed the correlation between differently expressed lncRNAs and mRNAs based on the Pearson correlation analysis. The x-axis and y-axis were the mRNA and lncRNA, respectively. Red indicated a strong relationship between lncRNAs and mRNAs and blue indicated weak ones based on the Pearson correlation coefficient. **b** The trans-regulation networks of lncRNA-mRNA were analyzed by using the maximum neighborhood component based on the Cytoscape software

The interaction of lncRNA and mRNA significantly affects the progression of many diseases, such as cancer and inflammation [33, 34]. It has been reported that the integrated network analysis of lncRNA-mRNA identifies the biomarkers in patients with OA of the knee [35]. Hence, we further explored the co-expression status of the above variant lncRNAs and mRNAs of OA in Tibetan and Chinese Han. Fundamentally, the Pearson correlation analysis showed the significant relationship of expression between the lncRNAs and mRNAs in Tibetan and Chinese Han samples, implying that the lncRNA-mRNA interaction may contribute to different clinical OA progression of Tibetans and Chinese Han (Fig. 3a) As we all know, lncRNA is able to modulate its targeted genes in the manner of cis-regulation and trans-regulation [36]. When the mRNA loci are within 300 k windows up- and downstream of the given lncRNAs, we can identify them as cis-regulated mRNAs of the corresponding lncRNAs [37]. Besides, lncRNAs involved in certain biological pathways may interact with transcription factors (TFs) [38]. The information of lncRNA-mediated *cis-* and *trans-*regulation is crucial for our understanding of RNA-mediated gene regulation and genome complexity [39]. Therefore, we tried to explore the cis-regulation and trans-regulation networks of lncRNA-mRNA in the system. Interestingly, we identified the top 100 significantly interacted lncRNA-mRNA in the trans-regulation analysis, in which the OTOA was the most remarkable gene (Fig. 3b).

**Fig. 4:**
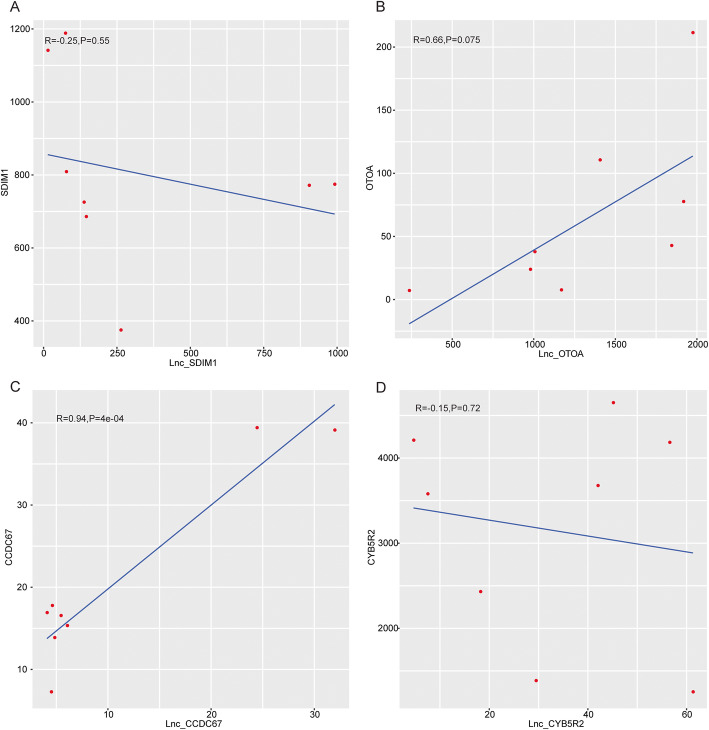
LncRNA-mRNA cis-regulation analysis demonstrates OTOA is the key factor of the variant lncRNA-mRNA networks of OA progression in Tibetan and Chinese Han. **a**–**d** The correlation of lnc-SDIM1 and SDIM1 **a**, lnc-OTOA and OTOA **b**, lnc-CCDC67 and CCDC67 **c**, and lnc-CYB5R2 and CYB5R2 **d** was shown. The x-axis and y-axis were the expression levels of lncRNA and mRNA, respectively. The *R* value was calculated by the fitted curve based on the linear regression, in which *R* value > 0 represented a positive regulation and *R* value < 0 indicated a negative regulation

Then, we further analyzed the lncRNA-mRNA cis-regulation networks based on the threshold of the Pearson correlation coefficient ≥ 0.7 and *P* value < 0.05. Surprisingly, we identified lnc-SDIM1 and SDIM1, lnc-CYB5R2 and CYB5R2, lnc-CCDC67 and CCDC67, and lnc-OTOA and OTOA with the Pearson correlation coefficients of 0.805, 0.833, 0.833, and 0.857, respectively (Fig. 4a, b). Moreover, lnc-OTOA and OTOA was the most significant lncRNA-mRNA relationship among these interactions (Fig. 4a, b). Taken together, we conclude that the OTOA, which was elevated in the cartilage tissues of Tibetans with OA compared to those in Chinese Han, was the most critical gene in the variant lncRNA-mRNA networks of OA progression in Tibetan and Han nationality. OTOA might be served as the potential biomarkers to characteristic the difference between the OA of Tibetans and Han nationality.

## Discussion

OA is the most frequent musculoskeletal disease, leading to functional decline and loss of quality of life [40]. Clinically, the disease is featured by joint pain, tenderness, crepitus, stiffness, limitation of movement with occasional effusion, and variable degrees of local inflammation [41]. The OA is mostly prevalent in the western regions of China, especially in Sichuan and Qinghai provinces and Tibet autonomous regions [42]. Despite various preventive and control measures, new cases still have been emerging in Tibet due to the specific living environment and life habits of Tibetan [43–45]. Unfortunately, the biological investigation and different processes of the development of OA between Tibetans and Han nationality is extremely limited and unclear. It has been reported that the single-nucleotide polymorphism (SNPs) rs7033979 in the ASPN gene may play a protective role in susceptibility to OA, and genes COL10A1 and HABP2 may play a role in the risk of developing OA in the Tibetan population [46, 47]. LncRNAs are involved in many diseases, and the exquisite cell type specificity of lncRNA function has clear implications for targeted therapy [48]. The differential expression of long noncoding RNAs by bioinformatics method in human knee OA may as novel dignosis biomarkers and therapeutic targets [49]. In addition, lncRNAs such as HOTAIR, MEG2, MALAT1, and DILC are involved in the modification of OA development [50–53]. In addition, recent studies have examined the expression of extracellular genomic material, including lncRNA and mRNA, which are aberrantly expressed in the body fluids of OA patients [54]. In this study, we identified a total of 117 lncRNAs and 297 mRNAs differently expressed in the cartilage tissues of Tibetans comparing with those of Chinese Han. We observed 49 lncRNAs and 158 mRNAs were upregulated, and 68 lncRNAs and 139 mRNAs were downregulated in the cartilage tissues of Tibetans. These data provide new evidence that the different modulation mechanisms may be involved in the Tibetan and Han nationality OA development. The differently expressed lncRNA and mRNA may be the discriminative biomarkers for the OA of Tibetan and Chinese Han.

Previous GO enrichment analysis showed that 119 upregulated genes were significantly enriched in blood vessel development, and KEGG pathway enrichment showed that genes were involved in the circadian rhythm pathway [55]. It has been reported that some risk pathways such as TGF-β signaling pathway and inflammatory bowel disease and risk biological processes such as chondrocyte differentiation and regulation of cartilage development are involved in the OA pathology [56]. Functional analysis of lncRNA in the synovial membrane of osteoarthritis patients revealed that lipopolysaccharide, angiogenesis, tumor necrosis factor (TNF) signaling, and mitogen-activated protein kinase (MAPK) signaling pathways might contribute to OA development [57]. Interestingly, our GO and KEGG analysis showed that several unreported processes such as muscle cell proliferation, cell-cell adhesion, and chemokine ligand 2 production and signaling pathways including Hippo signaling pathway, Wnt signaling pathway, and Ras signaling pathway were particularly identified in Tibetan OA patients compared to the Chinese Han OA patients. Our data revealed some different biological changes which were not similar to previous studies. These data suggest that the development of Tibetan and Chinese Han OA may feature particularly direct or indirect biological progression, and the specific mechanism is needed to further investigate.

It has been reported that the lncRNA-mRNA interaction is crucial for many pathological processes including OA [58]. CRNDE and LINC00152 play active roles in the development of OA [59]. The deregulated genes including USP46, CPVL, FKBP5, FOSL2, GADD45B, PTGS1, ZNF423, ADAMTS1, and TFAM might be involved in the pathology of OA [60]. Surprisingly, we demonstrated a significant relationship of expression between the lncRNAs and mRNAs in Tibetan OA samples compared to that of Chinese Han. It indicates that the c-expression of lncRNA and mRNA may be involved in the variant regulation mechanism of the OA progression of Tibetans and Han nationality. Strikingly, our trans-regulation analysis demonstrated the top 100 significantly interacted lncRNA-mRNA, in which the OTOA was the most remarkable gene. Moreover, the cis-regulation analysis further identified that lnc-OTOA and OTOA was the most significant lncRNA-mRNA relationship. These data imply that OTOA may play a critical role in the different lncRNA-mRNA networks between Tibetans and Han nationality OA progression. The effect of OTOA on the OA development of Tibetans and Han nationality and its molecular mechanism are needed to further explore. Moreover, it has been well-identified that microRNAs (miRNAs) play critical roles in the pathogenesis of OA by interacting with lncRNAs and various targeted genes from the aspects of diagnosis, physiopathology, and therapy [61]. It will be crucial and beneficial to investigate the interplay of lncRNA, miRNA, and mRNA networks in the development of OA, which may need to be explored further in the future. Furthermore, the different lncRNAs and mRNAs involved in multiple critical cellular biological processes and signaling identified in this study may be helpful for the exploration of diagnosis biomarkers for Tibetan OA patients. The significant lncRNA-mRNA association of lnc-OTOA and OTOA may serve as the potential diagnostic or therapeutic for the Tibetan OA patients in the clinic.

In conclusion, this study demonstrated that 117 lncRNAs and 297 mRNAs are differently expressed in Tibetan OA patients compared to the Chinese Han, which are involved in many characteristic biological processes and signaling pathways. LncRNA-mRNA co-expression analysis identified OTOA as the critical gene in the variant lncRNA-mRNA networks of OA progression between Tibetans and Han nationality. Our finding provides new insights into the differential lncRNA-mRNA scenario of OA development in Tibetan and Han nationality, improving the understanding of the discriminative mechanism of OA progression between Tibetan and Han nationality. Consequently, this should be helpful in shedding novel light on the development of nationality-specific therapeutic for OA.

## Supplementary Information

**Additional file 1: Table S1**

**Additional file 2: Table S2**

## Data Availability

The datasets used during the current study are available from the corresponding author on reasonable request. RNA was extracted from 3 Han nationality patients and 5 Tibetan patients with OA.

## Abbreviations

OA: Osteoarthritis
KEGG: Kyoto Encyclopedia of Genes and Genomes
KBD: Kashin Beck disease
PBS: Phosphate-buffered saline
MF: Molecular function
BP: Biological process
CC: Cellular component
PCC: Pearson correlation coefficient
TFs: Transcription factors
SNPs: Single-nucleotide polymorphisms
TNF: Tumor necrosis factor
MAPK: Mitogen-activated protein kinase
